# Study protocol for a feasibility randomised controlled trial of MOVE SMART—An intervention to increase physical activity, reduce sedentary behaviour and improve health outcomes in patients with psoriasis

**DOI:** 10.1371/journal.pone.0343922

**Published:** 2026-03-16

**Authors:** Gladys Onambele-Pearson, Ben Ives, Ishani Khosla, Rozemarijn Witkam, Matthew Roberts, Lucy Moorhead, Emma Bedson, Russ Cowper, Girvan Burnside, Helen S. Young

**Affiliations:** 1 Department of Sport and Exercise Sciences, Institute of Sport, Faculty of Science & Engineering, Manchester Metropolitan University, Manchester, United Kingdom; 2 Foundation Trainee, Royal Derby Hospital, University Hospitals of Derby and Burton NHS Foundation Trust, Derby, United Kingdom; 3 Division of Musculoskeletal and Dermatological Sciences, School of Biological Sciences, The University of Manchester, Manchester, United Kingdom; 4 The Dermatology Centre, Salford Royal Hospital, Manchester Academic Health Science Centre, The University of Manchester, Manchester, United Kingdom; 5 Liverpool Clinical Trials Centre, The University of Liverpool, Liverpool, United Kingdom; 6 St John’s Institute of Dermatology, Guy’s and St Thomas’ NHS Foundation Trust, King’s College London, London, United Kingdom; 7 Department of Health Data Science, Institute of Population Health, University of Liverpool, Liverpool, United Kingdom; PLOS: Public Library of Science, UNITED STATES OF AMERICA

## Abstract

Patients with psoriasis are less physically active than age-matched controls, due to psoriasis-specific barriers; significantly limiting their ability to benefit from health-promoting levels of physical activity. We co-designed an exercise intervention, with patients, which in proof-of-concept studies, led to significantly improved psoriasis; reduced cardiovascular disease/metabolic syndrome risk and enhanced wellbeing/psychosocial functioning. This suggested increased physical activity may improve health outcomes for those with psoriasis. However, individuals remained sedentary for prolonged periods, which also has a detrimental effect on health. We therefore developed a new intervention – MOVE SMART – to increase light-intensity physical activity and interrupt sedentary behaviour. This randomised controlled clinical trial (RCT) will assess the feasibility and acceptability of MOVE SMART. Utilising a decentralised, two-arm, trial design, people with Type 1 psoriasis with/without stable psoriatic arthritis, (n = 60) will be recruited from across the UK, and randomised to intervention (MOVE SMART with “Standard Care”, n = 30) or control (“Standard Care”, n = 30). The intervention group will follow MOVE SMART for 12-weeks, followed by activities of their own choice during weeks 13–24. MOVE SMART will prompt 2-minutes of light-intensity physical activity, following 30-minutes of continuous sedentary behaviour, during daytime waking hours. The study comprises of three Workstreams. In Workstream-1 participants will use wearable devices to allow monitoring of physical behaviour and adherence to MOVE SMART. A blood pressure monitor and body weight scales will be posted to participants for use throughout the study and functional capacity will be measured by video-link. At baseline, week-12 and −24 all participants will complete self-assessment of the extent/impact of psoriasis and wellbeing. Capillary blood will be collected using home-sampling kits. In Workstream-2 acceptability of the intervention will be evaluated and the trial design will be finalised in Workstream-3. The study is registered at www.isrctn.com (ISRCTN 17400289).

## Introduction

Sedentary behaviour [1] has a detrimental effect on health and wellbeing, independent of the amount of physical activity undertaken [2,3]. However, current physical behaviour guidelines, including those from the World Health Organisation (WHO) focus on achieving ≥150-minutes of physical activity per week or ~21-minutes/day. [4] This is despite acknowledgement of the limited efficacy and palatability of this approach in the general population [5] and barriers with long-term adherence [6]. Notably, these guidelines offer no clear recommendations on how to break up sedentary time [2,7]. Indeed, it is only at supramaximal levels of moderate-vigorous activities (≥420-minutes/week, ~ 60-minutes/day), that physical activity manages to offset the negative health effects of a high sedentary time, but such activity levels are not realistic for most [2,7].

Previously we identified that patients with psoriasis are less physically active compared to age-matched controls, due to psoriasis-specific barriers, [8] which significantly limits their ability to follow currently available exercise programmes and to benefit from health-promoting levels of physical activity [9]. Moreover, long-term health outcomes for people with psoriasis are poor and include depression, metabolic syndrome and cardiovascular disease (CVD) [10]. This presents a significant challenge to healthcare services, as more than 1.1 million people in the UK have psoriasis, an incurable, immune-mediated inflammatory disease, costing the UK National Health Service (NHS) over £500 million per annum [11,12].

Recognising the need to breakdown disease-specific barriers for this under-served population, we designed an incrementally progressive, light-intensity, walking intervention in partnership with patients with psoriasis [13] and measured the clinical impact of this in a proof-of-concept study [14]. Importantly each exercise session, which was led by a Sport and Exercise Scientist, was highly structured to ensure delivery of a pre-specified volume/dose of activity [13].

Promisingly, we observed significantly improved psoriasis control (including reduced Psoriasis Area Severity Index [PASI], p = 0.001 and ≥50% improvement in PASI [PASI-50] for 50% of participants [n = 16]; and reduced CVD/metabolic syndrome risk including reduced systolic and diastolic blood pressure (systolic: −8.5 mmHg, p = 0.004; diastolic: −3.0 mmHg, p = 0.01) [14]. We also observed enhanced wellbeing/psychosocial functioning and increased functional capacity [14]. However, although these data suggested that physical activity could constitute a promising therapeutic intervention for psoriasis, we observed no change in total sedentary time, as individuals continued to spend prolonged periods of time sitting or lying down, despite their increased levels of physical activity; potentially limiting the beneficial effect observed [14].

Having observed this in other studies we recognised that it is essential to consider both the amount and intensity of physical activity and the duration of sedentary time when assessing the overall physical behaviour profile of an individual [15]. Promisingly, breaking up sedentary time with short bouts of light-intensity physical activity offers a potential solution and we have found this approach acceptable in previous studies [16,17]. Others describe that it enhances cardio-metabolic health and physical function [18,19]. We therefore designed a new, low-cost, physical behaviour programme, MOVE SMART, to overcome the limitations of our pilot intervention by reducing and interrupting periods of sedentary behaviour, whilst increasing tolerable physical activity in patients with psoriasis. In this study we will assess the feasibility and acceptability of a randomised controlled trial (RCT) of MOVE SMART prior to investigating clinical efficacy across several critical health outcomes for patients with psoriasis in an appropriately powered multi-centre RCT.

## Materials and methods

**Fig 1:**
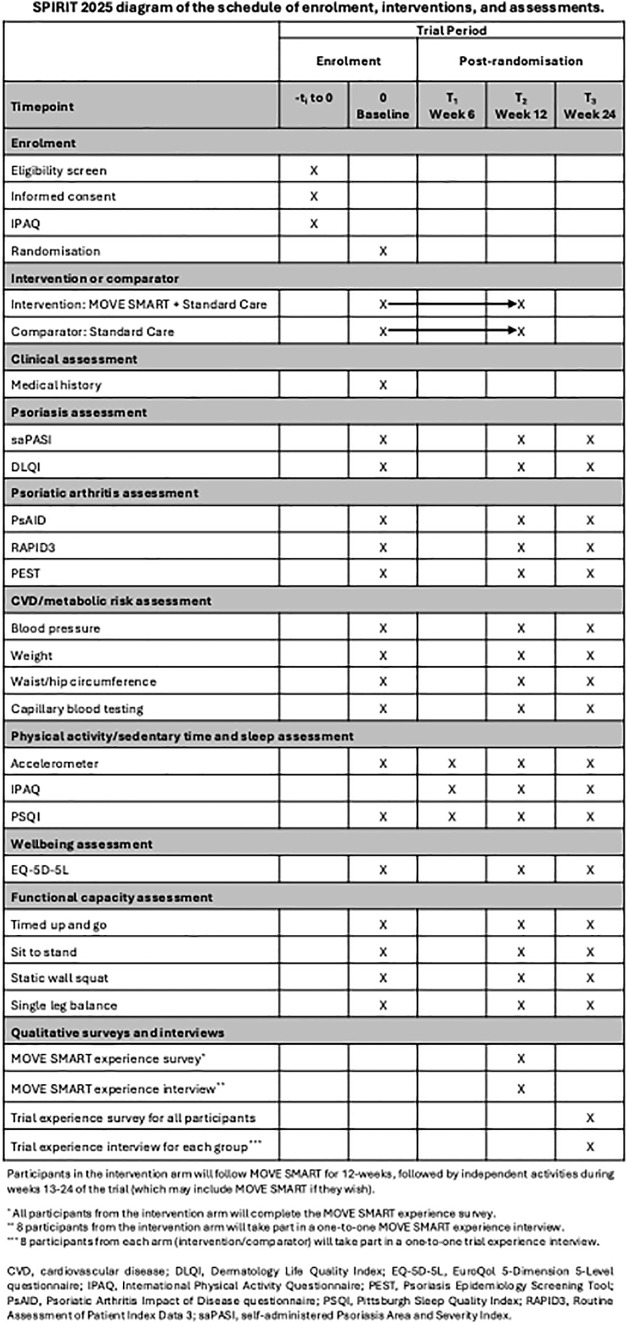
SPIRIT schedule of enrolment, interventions, and assessments for the MOVE SMART study.

**Fig 2:**
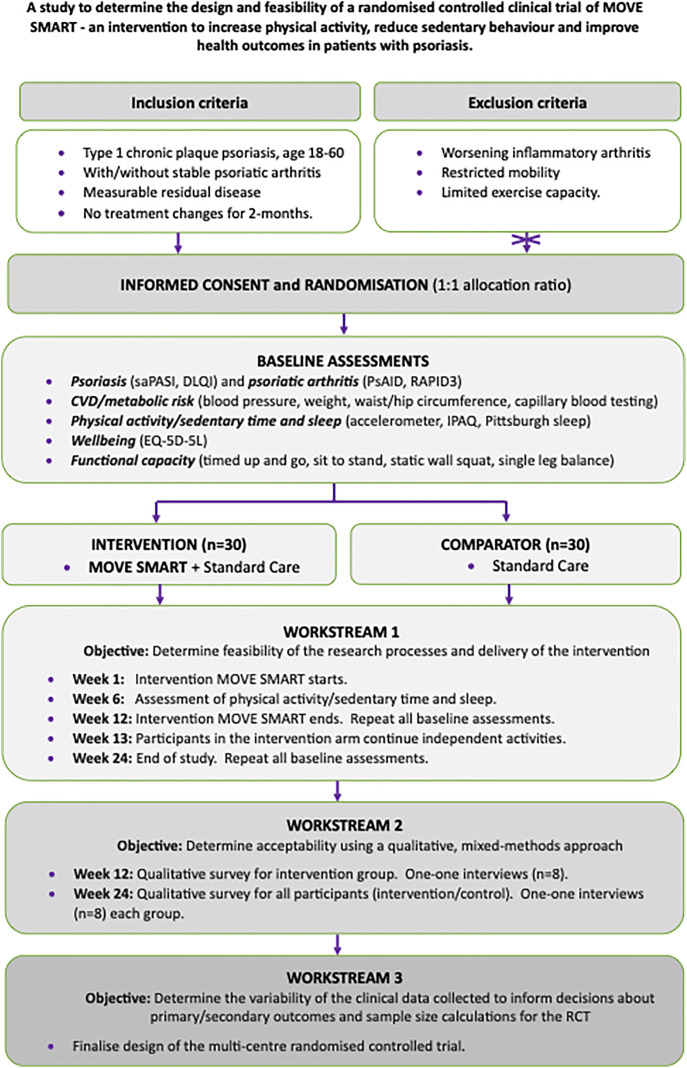
Comprehensive overview of the MOVE SMART study design.

The protocol for our decentralised, randomised controlled feasibility trial is reported in accordance with the Standard Protocol Items: Recommendations for Interventional Trials (SPIRIT) Checklist (S1 Fig) [20]. A SPIRIT schedule and overview of the study design can be found in (Figs 1 and 2). The study has been approved by the North-West – Greater Manchester West Research Ethics Committee, the Health Research Authority and Health and Care Research Wales (24/NW/0362), is sponsored by The University of Manchester and is registered at ISRCTN17400289. The study has been adopted onto the National Institute for Health and Care Research (NIHR) Research Delivery Network portfolio, given it was peer-reviewed, offered discernible value to the NHS and was deemed to be of high quality [21]. The study is registered with the NIHR Associate Principal Investigator Scheme, which aims to develop health and care professionals to become the Principal Investigators in the future [22].

### Research question

Does a novel physical behaviour intervention (developed with individuals who have psoriasis) have clinical utility in the management of psoriasis?

### Study design and recruitment

We will utilise a digitally enabled, remote trial delivery model to recruit 60 participants with Type 1 chronic plaque psoriasis (disease onset before age 40 years)**,** through self-referral of interested individuals (from across the UK) directly to the study team. This will be supported by the national reach of our partner organisations – the UK Psoriasis Association and the UK Dermatology Clinical Trials Network (UK DCTN) – who will advertise the study through their online, mailing and journal/newsletter platforms. Our study is also registered on the NIHR Be Part of Research Service, which enables potential volunteers to find and take part in vital health and care research across the UK [23].

As the UK’s leading national charity and member organisation for individuals with psoriasis, the UK Psoriasis Association regularly communicates with people from all over the country whose lives are affected by the condition enabling them to connect via their online forums (>20,000 registered users in 2023), through their website (507,359 visits in 2023) and social media (>50,000 followers). We will also benefit from the UK DCTN expertise and infrastructure, which includes support with recruitment via their diverse patient panel, and ongoing publicity for the study via the UK DCTN membership (>1000 patients/clinicians/other stakeholders with an interest in skin research).

### Sample size

Our sample size is calculated based on our quantitative traffic light progression criteria (Table 1). A sample size of 30 per group will give at leave 80% power to reject a true proportion being in the red zone (< 50%) if we observe a proportion in the green zone (≥ 75%) [24].

**Table 1:** MOVE SMART feasibility measures and progression criteria.

Criterion	Critical feasibility outcome	Green	Amber	Red
(1) Recruitment rate	Recruitment of 60 participants within a 12-month window (percentage of target recruited)	≥ 75%	50-74%	< 50%
(2) Retention rate	Percentage of participants remaining in the study until completion (24-weeks total)	≥ 75%	50-74%	< 50%
(3) Clinical assessments	Percentage of participants with available data on clinical assessments	≥ 75%	50-74%	< 50%
(4) Capillary-blood self-collection	Percentage of participants returning capillary-blood home-collection kits	≥ 75%	50-74%	< 50%
(5) Adherence to intervention	Percentage of participants completing the MOVE SMART intervention	≥ 75%	50-74%	< 50%
(6) Intervention and trial protocol	Percentage of participants report positive experiences (acceptability) and adherence to intervention components (feasibility)	≥ 75%	50-74%	< 50%
(7) Safety of intervention	Monitoring and review of intervention related serious adverse events (SAEs). The TSC will oversee SAEs across the intervention and control arms. GO: When the rates of intervention-related SAEs are low and the TSC is happy for continuation to a definitive trial. STOP: When the rates of intervention-related SAEs are considered unacceptably high, or when any single SAE indicates an unacceptable risk to participants.			

### Inclusion and exclusion criteria

Our population of interest includes those with Type 1 psoriasis (who have measurable residual disease), with or without stable psoriatic arthritis, aged 18–60 years (due to the prevalence of CVD disease in the > 60s in the UK, which means that we are likely to detect greater differences in CVD risk in those <60) [25]. Participants must have had no treatment or dose changes for 2-months prior to entering the study and have a high sedentary time (irrespective of level of physical activity): defined using the Short Form International Physical Activity Questionnaire (IPAQ) as >8 hours per day [26].

Exclusion criteria include being unable to rise from a sitting to a standing position, walk around a room (independently or aided by a walking stick, frame or trolly) whilst maintaining a steady pace (~30 steps/minute) for 2-minutes without stopping, having worsening inflammatory arthritis or significant comorbidity that would restrict adherence with the intervention. Those regularly engaging in HIGH levels of physical activity (as defined by IPAQ) whilst spending MINIMAL time in sedentary activities during day-waking hours (as calculated by IPAQ, < 6 hours per day) are also excluded from participation [26].

### Randomisation

Following written informed consent documented and witnessed by the study team, all those who fulfil the inclusion criteria will be randomized to either the intervention (MOVE SMART with “Standard Care”) arm or the control arm (“Standard Care”) of the study. We will use a stratified block randomisation procedure to ensure that participants in the intervention and control groups are balanced on age and sex. Importantly, Standard Care” for patients with psoriasis does NOT include advice on physical activity or sedentarism in the UK.

### The intervention – MOVE SMART

The purpose of MOVE SMART is to interrupt bouts of prolonged sedentary behaviour with light-intensity physical activity, thus reducing overall sedentary time. Its design is based upon two key points. First, WHO recommendations on physical activity together with our published data, gives a theoretical starting point for the amount of physical activity which may be beneficial [4,14,16,17,27]. Second, fragmentation of sitting time every 30-minutes over a 12-hour period (09:00–21:00), is based upon recent epidemiological evidence linking a more prolonged sedentary accumulation pattern (≥30-minute bouts) with greater all-cause mortality [28].

MOVE SMART will therefore be confined to a 12-hour period between 09:00 and 21:00 and will involve up to 24 2-minute bouts (48-minutes) of upright light-intensity physical activity throughout the day. An important aspect is that participants engage in light-intensity physical activities which are ADDITIONAL to their physical activity at baseline. Participants will utilise a free Mobile Application on their smartphone which we will customise to prompt physical activity following 30-minutes of sedentary behaviour. On receiving an alert participants will undertake 2-minutes of light-intensity physical activity and will perform a mixture of body weight only (such as walking around the home, office, or outdoors at a steady pace, side-to-side steps, Tai Chi movements) and aided-resistance work using resistance bands (Thera-bands) which will be supplied at the start of the study. Importantly, participants will have autonomy in selecting the specific type of physical activity they wish to follow. To enhance clarity of instruction, each recommended activity will be meticulously documented in a series of instructional videos, and a comprehensive illustration booklet will be disseminated via postal mail. Patient and Public Involvement (PPI) advisors have endorsed this approach as it will cater to participants with diverse learning preferences.

Participants in the intervention arm will follow MOVE SMART for the first 12-weeks, followed by independent activities during weeks-13–24 of the trial (which may include MOVE SMART if they wish). Feedback from PPI groups described similar interventions acceptable, easy to incorporate into a daily routine and suggesting that with time, participants became less reliant on device prompts, and were more able to adopt behaviour change: *“once you were familiar with how to use it, it became habit forming”, “I (attempted) to “beat” the prompt through self-regulating my own Sedentary Behaviour as I was now … standing during TV adverts”* [15–17,29].

For participants who do not have access to smartphones or tablets, we will provide these devices for the duration of the study. We will also provide mobile data plans or Wi-Fi access points to participants who lack reliable internet connectivity, which will ensure continuous access to the digital tools required for the study. We will monitor the need for these and will reflect on the implications for a larger trial.

We will also offer training sessions to all participants to improve their digital literacy, tailored to the needs of different demographic groups, including older individuals and those from lower socio-economic backgrounds. The research team will also assist participants with technical difficulties encountered throughout the study.

### Specific research objectives

1) Determine feasibility of the research processes (recruitment rate, willingness of eligible participants to be randomised, retention rate, reasons for loss to follow-up) and delivery of the intervention (fidelity of adherence with MOVE SMART, identify potential adverse effects and test collection of clinical and health-related outcome measures).
2) Determine acceptability of the intervention using a qualitative, mixed-methods approach.
3) Finalise the design of the future multi-centre RCT.
4) Inform decisions about primary and secondary outcomes for the RCT and associated sample size calculations.

Three Workstreams will deliver the scientific and patient-centred objectives:

#### Workstream-1: Testing feasibility of the research processes and delivery of MOVE SMART

A blood pressure monitor, tape measure and body weight scales will be posted to all participants for use throughout the study. Data collection methods will include those proposed for a full-scale multi-centre evaluation and involve face-face and/or virtual interaction with the study team (as per the participant’s preference and geographical location). Assessment of psoriasis, wellbeing, sleep, anthropometric variables and functional capacity will be made for those in both the intervention and control arms at baseline, week-12, and −24. Participants will complete a self-assessment (sa)PASI [30] and Dermatology Life Quality Index (DLQI) [31]; simple, self-administered tests of the extent/impact of psoriasis, which we have successfully used in previous studies [32]. Psoriatic arthritis will be assessed using the Psoriasis Epidemiology Screening Tool (PEST) Questionnaire [33] and Routine Assessment of Patient Index Data 3 (Rapid3) [34]. Wellbeing and sleep will be assessed using validated questionnaires, namely the EuroQol 5-Dimension 5-level questionnaire (EQ-5D-5L) [35] and the Pittsburgh Sleep Quality Index (PSQI) [36]. Participants will record their waist/hip circumference, body weight and blood pressure. They will also have assessment of functional capacity, which will be conducted by video-link (as in our previous studies) [13,14]. These data will provide indices of function and disease/health burdens against which changes in physical/sedentary behaviours can be assessed and interpreted.

Lancets, alcohol swabs, hypoallergenic dressings, and capillary blood collection tubes (Becton Dickson, BD Diagnostics, Plymouth UK) to enable home-collection (finger-prick) sampling of capillary blood, [37] will be mailed to all participants at baseline, week-12, and −24 timepoints. Training in home collection of capillary blood will be provided. The PPI co-applicant/public contributor and lived-experience advisors strongly welcomed this approach which they believed would reduce travel by participants for in-person venepuncture and lower the carbon footprint of our study.

Physical behaviour assessment will be made at baseline, week-6, −12, and −24. Prior to each time point a small, lightweight (~16 g, 4 x 1.3 cm) accelerometer (GeneActiv), provided by the study team will be posted to all participants. Accelerometers enable objective monitoring of physical activity/sedentary behaviour and sleep data to determine intervention fidelity and participant engagement, which were considered vitally important by the team’s PPI work. The choice to use the GENEActiv device, as a suitable tool, is based on these insights. In previous studies participants have reported excellent wearability (on the thigh or wrist) of the devices *“even … on holiday”*, finding them *“unobtrusive”* [15–17]. We have >10-years’ experience utilising these and have developed and validated algorithms to interrogate their outputs against laboratory-based calibrations of physical behaviour [14–17,27]. Accelerometers will be pre-programmed and paper/video-based instructions will explain how to secure/use the device (co-developed with the team’s PPI networks). Participants will leave the device in place for seven days, as in previous studies, to remove weekday/weekend activity differences and provide time for habituation to reduce changes in behaviour caused by awareness of being ‘observed’ [38]. Participants will return-post the accelerometer at the end of the collection period. Participants will also complete questionnaires designed to assess physical behaviour in people with health conditions, thus enabling self-reported and objective measurement of activity, sedentarism and sleep to be compared [8,9,30].

#### Workstream-2: Evaluating acceptability of MOVE SMART

A two-phase, mixed methods design will be used to generate in-depth insight into participants’ experiences and acceptability of: a) the MOVE SMART intervention, and b) the RCT protocol. Data collection materials (i.e., surveys and interview guides) were developed in consultation with the team’s PPI network through activities including pilot survey completion, annotated document feedback, and interactive discussion.

##### Phase one: Acceptability and user experience of the MOVE SMART intervention.

###### Mixed methods survey.

All participants allocated to the intervention arm (n = 30) will be invited to complete an online survey following the 12-week MOVE SMART intervention. The survey (S2 Fig) comprises Likert-scale items alongside open-ended responses focused on participants’ expectations of MOVE SMART, motivations for engagement, experiences of following the intervention, perceptions of training and support, perceived impacts on psoriasis and broader health outcomes, overall satisfaction, and recommendations for improvement [39]. The survey concludes with a question asking participants whether they would be willing to take part in a one-to-one interview to explore their experiences in greater depth.

###### One-to-one interviews.

will be conducted with a subsample of intervention participants (n = 8). The interviews will take place online and will last ~45-minutes. Participants will be purposively sampled to reflect diversity in demographic characteristics (e.g., gender, age, ethnicity) and variation in experiences reported within the survey. Interviews will be digitally recorded and transcribed verbatim. A semi-structured topic guide (S3 Fig) will be used to direct interaction while allowing flexibility for open discussion of opinions, perspectives, and experiences. These interviews will allow for elaboration and clarification of survey findings, as well as offering an opportunity to generate new insight into the MOVE SMART user experience [40].

##### Phase Two: Acceptability of trial processes and participation in the feasibility RCT.

###### Mixed methods survey.

All participants in both the intervention (n = 30) and control (n = 30) arms will be invited to complete an online survey following completion of the 24-week RCT. The survey (S4 Fig) includes Likert-scale items and open-ended responses examining participants’ motivations for trial participation, experiences of the trial processes, and suggestions for improvement. For participants in the intervention arm, the survey will also explore physical activity behaviours during weeks 13–24, to assess if, how, and why intervention participants have (or have not) continued to adopt lifestyle behaviours aligned with the MOVE SMART intervention [39]. The survey for both groups concludes with a question asking whether they would be willing participate in a one-to-one interview to discuss their experiences in greater detail.

###### One-to-one interviews.

A purposive sub-sample of participants from both the intervention (n = 8) and control (n = 8) arms will be invited to take part in an online, ~ 45-minute one-to-one interview. Sampling will be informed by demographic characteristics and diversity of trial experiences reported in the survey. Interviews will be digitally recorded and transcribed verbatim. A semi-structured interview guide (S5 Fig) will be used to provide focus while retaining flexibility for free discussion of opinions, perspectives, and experiences. These interviews will add depth and nuance to understanding participants’ experiences of trial participation and trial procedures [40].

**Workstream-3: Finalise design of the MOVE SMART RCT.**

The purpose of Workstream-3 is to investigate the feasibility of progression to a definitive multi-centre RCT and to highlight any potential issues that may need to be addressed to improve delivery. Specifically, feasibility outcomes of interest include recruitment of participants, completeness of outcome measures, and adherence to the intervention MOVE SMART. These will be assessed in each arm separately, as there may be systematic differences between them; for example, those randomised to the control arm may be less likely to remain engaged than those randomised to the MOVE SMART arm.

The decision to progress to full trial will be based on a traffic light system with the feasibility objectives and measures used as pre-defined stop/amend/go criteria as specified in (Table 1). Criteria meeting all “go” (green) outcomes would lead to progression to a full RCT and no or minor revisions would be required. However, if criteria meet “amend” (amber) targets, reasons for this will be investigated with an aim to identify aspects amenable to change. One or more amber outcomes would suggest that alterations or adaptations to the trial protocol, assessments, or intervention, would be required. These, in turn would be supported by the qualitative work stream and discussed with the Project Management Group (PMG). If criteria meet “stop” (red) targets, reasons will be analysed and discussion within the PMG and with independent oversight committees as major alteration would be required before conducting a full RCT. If it is determined that these rates cannot be improved, then a full trial will not be recommended.

In addition, having collected qualitative data on the acceptability of the trial procedures and intervention in Workstream-2, we will draw on these perspectives with a view to their incorporation or refinement prior to finalising the design of the definitive RCT. We will monitor participation and engagement metrics to identify any disparities related to digital access [41]. We will collect qualitative feedback from participants to understand their experiences and challenges with the digital tools. Based on our findings, we will adapt our approach in a future larger clinical trial, to address any identified issues and improve delivery and equity. Heterogeneity within the population will also be considered and whether the intervention’s feasibility and effectiveness may differ by demography will be examined. It is important to us to ensure inclusivity of participation and that there are no barriers to participation by individuals, caused by gender, ethnicity, digital poverty or other, as yet unforeseen factors, in a definitive trial. We will report on adverse events, although anticipate no issues that are likely to be detrimental to participants. Safety monitoring will be performed by the Trial Steering Committee (TSC).

We will finalise the intervention specification costs, and research procedures based on our feasibility and acceptability testing.

### Statistical analysis

Analyses will be completed at the end of the study using well-validated statistical packages. Reporting will follow the CONSORT guidance for pilot and feasibility studies, and a CONSORT flow diagram will display data from screening, recruitment and follow-up enabling estimation of eligibility, recruitment, consent and follow-up rates [42]. Analysis will be on an intention-to-treat principle as far as is practically possible and focussed on assessing the criteria for deciding whether to progress to a full trial. All estimates of proportions will be presented with 95% confidence intervals. Rates of recruitment and attrition will be presented for participants, along with the proportion of MOVE SMART interventions which are successfully delivered. The proportion of missing data in the proposed trial outcome measures will be assessed. Exploration of estimates of efficacy will involve a group-wise comparison of the primary outcome: percentage of participants achieving PASI-50 (≥50% improvement of PASI compared to baseline, considered clinically meaningful) [43] as measured at week-12 by completion of (sa)PASI. No formal testing of intervention effect will be carried out but estimates of differences between outcome measures for the MOVE SMART and control groups will be presented, with 95% confidence intervals, to assess whether a clinically important improvement in outcome would be plausible in a full trial.

Likert-scale survey data will be analysed descriptively using frequency distributions to illustrate how often each response category occurred. Qualitative data generated from open-ended survey responses and one-to-one interviews will be analysed using Reflexive Thematic Analysis (RTA), following six iterative phases: familiarisation with the data; coding; generating initial themes; developing and reviewing themes; refining, defining, and naming themes; and writing up [44,45]. Developing thematic interpretations will be shared with the team’s PPI network to invite reflective input and enhance the quality and relevance of findings.

### Trial status

The study is actively underway in accordance with protocol version 3, dated December 12, 2024 (S1 File). Recruitment of participants started on February 1, 2025, and will be completed by July 31, 2026. The intervention phase and the last follow-up assessments will be completed by October 2026. The study is currently progressing according to the planned timeline. Data collection, analysis and the generation of meaningful results is expected to be completed by December 2026.

## Discussion

The WHO recognises psoriasis, an immune-mediated inflammatory disease affecting up to 3% of the UK population, as “a serious non-communicable disease” and the imperative to address the “consequences of psoriasis” and “the exclusion of patients from healthcare settings and daily life” [46]. We have previously identified that patients, because of their psoriasis, are less likely to engage in physical activities and are restricted in accessing leisure facilities and our current study seeks to redress this. Indeed, our proof-of-concept work suggests that coupling low- cost, adjuvant, physical activity strategies with standard therapies for psoriasis, could improve disease outcomes and reduce significant additional health burdens such as depression, CVD and metabolic syndrome [14].

Importantly this study champions the *“no decision about me, without me”* ethos. Specifically, we utilised facilitated focused group discussions with lived experience contributors, including the PPI Lead (RC), to inform all aspects of the design of our preliminary work, including the physical activity intervention utilised in our pilot study. *“Inclusivity”* emerged as a key theme during our design workshops with patients who advocated that the intervention should be *“free”*, have *“measurable health benefits”* and foster a *“safe environment free of stigmatisation”* for participants [13]. The voices and insights of individuals with lived experience of psoriasis have been integral to the development of every aspect of the current study and the active involvement of public contributors throughout all stages of project design. In addition, significant input from the UK DCTN, including their patient panel (40 members in total) and wider membership (>1000 patients/clinicians/other stakeholders with an interest in skin research), has been crucial in refining the study protocol. Moreover, the trial responds to the most important research priorities identified by healthcare professionals and those with lived experience of psoriasis and psoriatic arthritis in the Psoriasis PSP [47] and the more recent Psoriatic Arthritis PSP; [48] collaborations facilitated by the James Lind Alliance and directly involving both the Trial Lead (HY) and the PPI Lead (RC).

As a research team that embodies gender, black and minority ethnic diversity, we recognise the critical importance of using evidence-based strategies to increase research participation in those from diverse ethnic groups, deprived backgrounds, and disabilities. Psoriasis is unequally distributed across geographical regions with higher prevalence reported in white people compared with other ethnic groups. Indeed, a 10-fold greater incidence of psoriasis is reported in populations from Western Europe compared to those from South-East Asia [49]. Barriers to minority participation in clinical trials are known to be multifactorial, but often include mistrust of the medical community [50]. We are therefore working through trusted organisations to ensure that diversity and inclusion are integral components of all our decision-making processes.

In conclusion, coupling physical behaviour strategies (that increase physical activity and reduce sedentarism) with standard therapies for psoriasis could improve disease outcomes and reduce the additional health burdens of depression, CVD and metabolic syndrome for patients with psoriasis.

## Supporting information

S1 File MOVE SMART clinical trial protocol. (PDF)

S1 Fig SPIRIT 2025 Checklist. (TIFF)

S2 Fig MOVE SMART experience survey. (TIFF)

S3 Fig MOVE SMART experience interview guide. (TIFF)

S4 Fig Clinical trial experience survey. (TIFF)

S5 Fig Clinical trial experience interview guide. (TIFF)
