# Supplementary material for: Study protocol for a feasibility randomised controlled trial of MOVE SMART—An intervention to increase physical activity, reduce sedentary behaviour and improve health outcomes in patients with psoriasis
Source: PLoS One. 2026 Mar 16;21(3):e0343922. doi: 10.1371/journal.pone.0343922 (PMC12991228; doi:10.1371/journal.pone.0343922)
Supplement: S1 File — (PDF) [file pone.0343922.s001.pdf]

RESEARCH PROTOCOL

RESEARCH FOR PATIENT BENEFIT (RFPB) PROGRAMME: NIHR207157:
A study to determine the design and feasibility of a randomised controlled clinical trial of MOVE SMART - an intervention to increase physical activity, reduce sedentary behaviour and improve health outcomes in patients with psoriasis.

Contents

1)	RESEARCH TEAM & KEY CONTACTS	3
2)	INTRODUCTION	5
3)	BACKGROUND	5
4)	STUDY OBJECTIVES	6
5)	STUDY DESIGN & PROTOCOL	7
6)	STUDY PARTICIPANTS	11
7)	OUTCOME MEASURES	12
8)	DATA COLLECTION, SOURCE DATA AND CONFIDENTIALITY	13
9)	STATISTICAL CONSIDERATIONS	15
10)	MONITORING AND QUALITY ASSURANCE	15
11)	SAFETY CONSIDERATIONS AND ADVERSE EVENTS	15
12)	PEER REVIEW	16
13)	ETHICAL and REGULATORY CONSIDERATIONS	16
14)	STATEMENT OF INDEMNITY	17
15)	FUNDING and RESOURCES	17
16)	PUBLICATION POLICY	17
17)	REFERENCES	19

1) RESEARCH TEAM & KEY CONTACTS

Chief Investigator:<br><br>Name: Dr Helen Young<br><br>Address:<br>The University of Manchester,<br>FBMH, School of Biological Sciences,<br>Division of Musculoskeletal and Dermatological Sciences,<br>Salford Royal NHS Foundation Trust,<br>M6 8HD<br><br>Email: helen.s.young@manchester.ac.uk	***Co-investigators:***<br><br>Name: Professor Gladys Pearson<br><br>Address:<br>Faculty of Science & Engineering<br>Department of Sport and Exercise Sciences<br>Manchester Metropolitan University<br>Institute of Sport Building<br>99 Oxford Road<br>Manchester<br>M1 7EL<br><br>Email: g.pearson@mmu.ac.uk
Co-investigators:<br><br>Name: Dr Ben Ives<br><br>Address:<br>Faculty of Science & Engineering<br>Department of Sport and Exercise Sciences<br>Manchester Metropolitan University<br>Institute of Sport Building<br>99 Oxford Road<br>Manchester<br>M1 7EL<br><br>Email: b.ives@mmu.ac.uk	***Co-investigators:***<br><br>Name: Dr Emma Bedson<br><br>Address:<br>Liverpool Clinical Trials Centre<br>University of Liverpool<br>2nd Floor<br>Institute in the Park<br>Alder Hey Children's NHS Foundation Trust<br>Eaton Road<br>Liverpool<br>L12 2AP<br><br>Email: ebedson@liverpool.ac.uk
Co-investigators:<br><br>Name: Dr Girvan Burnside<br><br>Address:<br>Liverpool Clinical Trials Centre<br>University of Liverpool<br>2nd Floor<br>Institute in the Park<br>Alder Hey Children's NHS Foundation Trust<br>Eaton Road<br>Liverpool<br>L12 2AP<br><br>Email: g.burnside@liverpool.ac.uk	***Co-investigators:***<br><br>Name: Mr Russell Cowper<br><br>Address:<br>The University of Manchester<br>FBMH, School of Biological Sciences<br>Division of Musculoskeletal and Dermatological Sciences<br>Salford Royal NHS Foundation Trust<br>M6 8HD<br><br>Email: rascalrussky@googlemail.com

Sponsor:	***Lead R&D Trust contact:***
Name: The University of Manchester<br><br>Sponsor contact: Ms Lynne Macrae, Faculty Research Practice Governance Coordinator<br><br>Address:<br>Faculty of Biology, Medicine and Health<br>4.64 Simon Buiding<br>University of Manchester<br>M13 9PL<br><br>Email: FBMHethics@manchester.ac.uk<br><br>Telephone: 0161 275 5436	Name: Katie Doyle<br><br>Address:<br>Northern Care Alliance NHS Foundation Trust,<br>Salford Royal Hospital<br>Summerfield House, 1st Floor<br>554 Eccles New Road<br>Salford<br>M5 5AP<br><br>Email: RDResearch@srft.nhs.uk

2) INTRODUCTION

People with psoriasis are less active than others. Psoriasis can lead to heart disease and other conditions. Being more active could prevent this. So, we created an exercise programme for people with psoriasis. Patients with psoriasis helped us do this. We found, being more active improved psoriasis and reduced the risk of heart disease. Patients found their wellbeing and physical ability improved too. But people were still spending long periods sitting/lying down. As this is also damaging to health, we developed MOVE SMART. MOVE SMART will help people be more active AND spend less time sitting down.

MOVE SMART is a new lifestyle programme for patients with psoriasis. We want to find out if MOVE SMART helps psoriasis and other conditions linked with psoriasis. To do that, we need to do a clinical trial. This study will find out whether a clinical trial is possible, or not.

The study will last 24-weeks. All volunteers will continue with their usual treatment and have an equal chance of being in the MOVE SMART group. MOVE SMART will last for 12-weeks. Volunteers will do activities of their own choice during weeks-13-24. MOVE SMART will prompt 2-minutes of activity (only) if people have spent 30-minutes sitting/lying down. In Workstream [1] volunteers will wear a small monitor (on their wrist/thigh). This will measure activity and sitting time. At Week-1, week-12 and -24 all volunteers will self-assess their psoriasis and wellbeing. The study team will help with this. We will collect blood at these time points using finger-prick kits. Blood pressure and body weight will also be recorded. We will measure physical ability. In Workstream [2] we will find out what people think about MOVE SMART. We will finalise our proposed clinical trial in Workstream [3].

The study is funded by the NIHR. Recruitment is through expression of interest directly to the research team.

3) BACKGROUND

Sedentary behaviour has a detrimental effect on health and wellbeing independent of the amount of physical activity undertaken. Previously we identified that patients with psoriasis are less physically active compared to age-matched controls, due to psoriasis-specific barriers, which significantly limits their ability to follow currently available exercise programmes and to benefit from health-promoting levels of physical activity. Moreover, long-term health outcomes for people with psoriasis are poor and include depression, metabolic syndrome and cardiovascular disease (CVD). This presents a significant challenge to healthcare services as more than 1.1 million people in the UK have psoriasis, an incurable, immune-mediated inflammatory disease, costing the NHS over £500 million per annum.

Recognising the need to breakdown disease-specific barriers for this under-served population, we designed a light-intensity, exercise intervention in partnership with patients with psoriasis and measured the clinical impact of this in a proof-of-concept study. Promisingly, we observed significantly: improved psoriasis control; reduced CVD/metabolic syndrome risk; enhanced wellbeing and psychosocial functioning. These data suggested that physical activity may have utility in the management of psoriasis. However, each exercise session, which was led by a Sport and Exercise Scientist, was highly structured to ensure delivery of a pre-specified volume/dose of activity which would limit roll out and delivery at scale. In addition, we observed no change in total sedentary time, as individuals continued to spend prolonged periods of time sitting or lying down, despite their increased levels of physical activity. We have observed this in other studies and recognise that it is essential to consider both the amount and intensity of physical activity and the duration of sedentary time when assessing the overall physical behaviour profile of an individual. Therefore, the aim of the current study is to assess the feasibility of a randomised controlled clinical trial (RCT) of a new physical behaviour intervention MOVE SMART, which overcomes the limitations of our pilot intervention by reducing and interrupting periods of sedentary behaviour (factors that are independently related to negative health outcomes), whilst increasing tolerable physical activity in patients with psoriasis.

This research is critically important because:

1. The utility of lifestyle/behaviour change and non-drug treatments to manage psoriatic disease were identified as the MOST important research priorities by healthcare professionals and those with lived-experience of psoriasis and psoriatic arthritis in the Psoriasis Priority Setting Partnership (PSP) and the

more recent Psoriatic Arthritis PSP; collaborations facilitated by the James Lind Alliance and directly involving both the Lead-Applicant and the Patient and Public Involvement (PPI) co-applicant.
2. The World Health Organisation (WHO) declared psoriasis "a serious non-communicable disease", and identified a need to address the "consequences of psoriasis" and "the exclusion of patients from healthcare settings and daily life". Our work has a) identified that patients, because of their disease, are less likely to engage in physical activities and are restricted in accessing leisure facilities and b) seeks to redress this.
3. Our proof-of-concept work suggests that coupling physical activity strategies with standard therapies for psoriasis could improve disease outcomes and reduce the additional health burdens of depression, CVD and metabolic syndrome.
4. This is a low cost, adjuvant management strategy, which is potentially beneficial across several critical health outcomes for patients with psoriasis (3% of the UK population).

Current recommendations from the WHO are to undertake physical activity (⩾150-minutes/week, ~21-minutes/day), despite acknowledgement of the limited efficacy and palatability of this approach in the general population and barriers with long-term adherence. However, WHO guidance offers no clear direction vis-a-vis breaking up sitting time, even though this has a highly negative effect on health outcomes independent of the amount of physical activity undertaken. It is only at supramaximal levels of moderate-vigorous activities (⩾420-minutes/week, ~60-minutes/day), that physical activity manages to offset the negative health effects of a high sedentary time, but such activity levels are not realistic for most.

Pilot data: Having identified that physical activity is important for cardiovascular health in patients with psoriasis and that psoriasis significantly limits the ability of sufferers to exercise and to benefit from health-promoting levels of physical activity, we designed an incrementally progressive, light-intensity, exercise intervention in partnership with patients with psoriasis. Importantly we dosed the volume of exercise delivered in each Sport and Exercise Scientist-led session meticulously, observing significantly: improved psoriasis control (including reduced Psoriasis Area Severity Index [PASI], p=0.001 and ⩾50% improvement in PASI [PASI-50] for 50% of participants [n=16]); and reduced systolic and diastolic blood pressure (systolic: -8.5 mmHg, p=0.004; diastolic: -3.0 mmHg, p=0.01). We also demonstrated significantly: enhanced wellbeing/psychosocial functioning and increased functional capacity. However, we observed no change in total sedentary time. These data suggested that physical activity could constitute a promising therapeutic intervention for psoriasis. However, we recognised the need to redesign our intervention in order to: i) reduce total daily sedentary time (which has a detrimental effect on health, independent of the amount of physical activity undertaken), ii) simplify roll out and delivery at scale and iii) facilitate investigation in an appropriately powered multi-centre RCT.

Promisingly, breaking up sedentary time with short bouts of light-intensity physical activity offers a potential solution and we have found this approach acceptable in previous studies. Others describe that it enhances cardio-metabolic health and physical function. In this proposal we will explore the feasibility of a RCT of a novel, low-cost, intervention which will i) increase light-intensity physical activity, ii) reduce overall sedentary time, and iii) interrupt bouts of prolonged (⩾30-minutes) sedentary behaviour; whilst not requiring change in pre-existing daily moderate-to-vigorous physical activity, an activity level that is particularly challenging for patients with psoriasis.

This study is funded by a grant from the NIHR Research for Patient Benefit programme: NIHR207157

4) STUDY OBJECTIVES

4.1 Primary Question/Objective:

Specifically, the following primary research question will be addressed:
-Does a novel physical behaviour intervention (developed with individuals who have psoriasis) have clinical utility in the management of psoriasis?

4.2 Secondary Question/Objective:

Specifically, the following secondary research objectives will establish the:
-feasibility of the research processes and delivery of the intervention

-acceptability of the intervention
-design and research outcomes for a subsequent multi-centre randomised controlled clinical trial

5) STUDY DESIGN & PROTOCOL

5.1 Participants

This project will involve male and female patients with Type 1 (disease onset before age 40 years) with/without stable psoriatic arthritis, aged 18-60 years (due to the prevalence of CVD disease in the >60s in the UK, which means that we are likely to detect greater differences in CVD risk in those <60).[1]

Participants (n=60) will be randomised to intervention or control arms of the study.

Intervention: "Standard Care" for patients with psoriasis does NOT include advice on physical activity or sedentarism.[2] Therefore, eligible participants will be randomised to MOVE SMART in addition to "Standard Care" (n=30).

Control: Participants in the control group will receive "Standard Care" (n=30) only.

5.2 Study Intervention and/or Procedures

Participants (n=60) will be randomised in to either the intervention ("Standard Care" + MOVE SMART) arm or the control arm ("Standard Care") of the study.

ALL STUDY ACTIVITIES WILL TAKE PLACE VIRTUALLY.
CONSENT AND DATA COLLECTION (VIA SELF-COMPLETION QUESTIONNAIRES AND MEDICAL HISTORY FORMS) WILL BE COMPLETED ONLINE USING THE QUALTRICS PLATFORM WHICH IS APPROVED BY THE UNIVERSITY OF MANCHESTER.
FINGER-PRICK CAPILLARY BLOOD WILL BE COLLECTED BY VOLUNTEERS AT HOME AND RETURNED (VIA MAIL) DIRECTLY TO THE RESEARCH TEAM AT THE UNIVERSITY OF MANCHESTER (see section 8 for further details on collection, transfer, storage and analysis of these samples).

MOVE SMART: The purpose of MOVE SMART is to interrupt bouts of prolonged sedentary behaviour with light-intensity physical activity, thus reducing overall sedentary time. Its design is based upon two key points. First, WHO recommendations on physical activity together with our published data gives a theoretical starting point for the amount of physical activity that may be beneficial.[3-7] Second, fragmentation of sitting time every 30-minutes over a 12-hour period (09:00-21:00), is based upon recent epidemiological evidence linking a more prolonged sedentary accumulation pattern (⩾30-minute bouts) with greater all-cause mortality.[8] MOVE SMART will be confined to a 12-hour period between 09:00 and 21:00 and will involve up to 24 2-minute bouts (48-minutes) of upright light-intensity physical activity throughout the day. An important aspect is that participants engage in light-intensity physical activities which are ADDITIONAL to their physical activity at baseline. Participants will utilise a free Mobile Application on their smartphone which we will customise to prompt physical activity following 30-minutes of sedentary behaviour. It is important to note that the Application will ONLY alert when/if a participant remains sedentary (without moving) for 30-minutes or more. The advantage of this Application over other prompting devices is that it can be programmed to avoid alarm fatigue (borne out of iterative testing by our PPI advisors), which could lead to participants ignoring movement prompts and negatively affect intervention compliance. On receiving an alert participants will undertake 2-minutes of light-intensity physical activity and will perform a mixture of body weight only (such as walking around the home, office, or outdoors at a steady pace, side-to-side steps, Tai Chi movements) and aided-resistance work using resistance bands (Thera-bands) which will be supplied at the start of the study. Importantly, participants will have autonomy in selecting the specific type of physical activity they wish to follow. To enhance clarity, recommended activities will be documented in an illustrated booklet, which will be sent to participants. Our PPI advisors have endorsed this approach as it will cater to participants with diverse learning preferences. Participants in the intervention arm will follow MOVE SMART for the first 12-weeks, followed by independent activities during weeks-13-24 of the trial (which may include MOVE SMART if they wish). Feedback from PPI groups described similar interventions acceptable, easy to incorporate into

a daily routine and suggesting that with time, participants became less reliant on device prompts, and were more able to adopt behaviour change: “once you were familiar with how to use it, it became habit forming”, “I (attempted) to “beat” the prompt through self-regulating my own Sedentary Behaviour as I was now … standing during TV adverts”.[5-6,9-10]

For participants who do not have access to smartphones or tablets, we will provide devices for the duration of the study, with secured funds available to support this on an “an-needed” basis. This ensures that all participants can utilize the MOVE SMART mobile application and related digital tools without financial burden. We will also provide mobile data plans or Wi-Fi access points to participants who lack reliable internet connectivity. This will ensure continuous access to the digital tools required for the study. We will monitor the need for these and will reflect on the implications for a larger trial.
We will also offer training sessions to all participants to improve their digital literacy, tailored to the needs of different demographic groups, including elderly individuals and those from lower socio-economic backgrounds. The recruited PDRA will be available to assist participants with technical difficulties throughout the study.

Three workstreams will deliver the scientific and patient-centred objectives:
Workstream [1]: Testing feasibility of the research processes and delivery of MOVE SMART.
A blood pressure monitor, tape measure and body weight scales will be posted to all participants for use throughout the study. Data collection methods will include those proposed for a full-scale multi-centre evaluation and involve face-face and/or virtual interaction with the study team (as per the participant’s preference and geographical location). Assessment of psoriasis, wellbeing, sleep, anthropometric variables and functional capacity will be made for those in both the intervention and control arms at baseline, week-12, and -24. Participants will complete a (sa)PASI and dermatology life quality index; simple, self-administered tests of the extent/impact of psoriasis, which we have successfully used in previous studies.[11] Wellbeing and sleep will be assessed using validated questionnaires.[12] Participants will record their waist/hip circumference, body weight and blood pressure. They will also have assessment of functional capacity, which will be conducted virtually (as in our previous studies).[3,13] These data will provide indices of function and disease/health burdens against which changes in physical/sedentary behaviours can be assessed and interpreted.

Lancets, alcohol swabs, hypoallergenic dressings, and capillary blood collection tubes (Becton Dickson, BD Diagnostics, Plymouth UK) to enable home-collection (finger-prick) sampling of capillary blood,[14] will be mailed to all participants at baseline, week-12, and -24 timepoints. Training in (home-based) capillary blood collection will be provided. Our PPI co-applicant/public contributor and lived-experience advisors strongly welcomed this approach which they believed would reduce travel by participants for in-person venepuncture and lower the carbon footprint of our study. We have chosen to adopt the user-friendly methodology developed by Imperial College London who report exceptional feasibility, reliability, and acceptability of the kits with over 85% of users describing the blood collection “easy”.[14] Critically analytes from capillary blood sampled in this way were comparable to those collected by standard venepuncture.[14] We will evaluate whether home-collection of capillary blood offers an acceptable/feasible alternative to standard venepuncture in later Workstreams as investigation of lipid/metabolic markers would be included in the subsequent RCT. Analysis of lipid/metabolic/biochemical markers from the samples collected here will be conducted using ELISA.

Physical behaviour assessment will be made at baseline, week-6, -12, and -24. Prior to each time point a small, lightweight (~16 g, 4 x 1.3 cm) accelerometer (GeneActiv), provided by the study team will be posted to all participants. Accelerometers enable objective monitoring of physical activity/sedentary behaviour and sleep data to determine intervention fidelity and participant engagement, which were considered vitally important by our PPI work. Our choice of the GENEActiv device, as a suitable tool, is based on these insights. We have >10-year experience utilising these and have developed and validated algorithms to interrogate their outputs against laboratory-based calibrations of physical behaviour.[3,5-7,9,13,15-16] Accelerometers will be pre-programmed and paper/virtual instructions will explain how to secure/use the device (co-developed with our PPI networks). Participants will leave the device in place for seven days, as in previous studies, to remove weekday/weekend activity differences and provide time for habituation to reduce changes in behaviour caused by awareness of being ‘observed’.[3,7,13,17] Participants will return-post the accelerometer at the end of the collection period. Participants will also

complete questionnaires designed to assess physical behaviour in people with health conditions, thus enabling self-report and objective comparison of activity, sedentarism and sleep.[12,15-16]

Workstream [2]: Evaluating acceptability of MOVE SMART.
The purpose of this workstream [2] is to examine the participants' experiences and acceptability of a) the MOVE SMART intervention and b) the feasibility randomised control trial. Data will be gathered through a two-phased, multi-method qualitative design.

Phase one: will explore the user experience of the MOVE SMART intervention. All participants from the intervention arm (n=30) will be invited to complete an online, qualitative survey following 12-weeks of MOVE SMART (during week-13). Qualitative surveys are a proven, yet underutilised, method for producing rich, focused, and complex accounts of participants' subjective experiences, perspectives, and practices.[18] The survey was constructed by the research team in consultation with our PPI network. The survey questions will centre on the user experience of MOVE SMART (including enablers and barriers for participation, likes and dislikes, expectation versus reality). This will also permit evaluation of the impact of digital poverty and whether there is digital inequality as a result of limited access to prerequisite infrastructure (such as Smartphones, Wi-Fi, or computers) or a lack of digital skills, the latter increasingly recognised as a public health concern.[19] Finally, participants will be asked whether they wish to participate in a follow-on one-one semi-structured interview with a member of the research team (see below). Data will be analysed using Reflexive Thematic Analysis (RTA), which involves six iterative phases: familiarising yourself with the dataset; coding; generating initial themes; developing and reviewing themes; refining, defining, and naming themes; and writing up.[20] RTA is widely used in sport, health, and exercise research, providing a robust method for developing deep and compelling understanding of patterned meaning across a qualitative dataset.[21] As part of our RTA approach, findings from the survey will be shared with our PPI networks and comments invited regarding our developing thematic interpretations.

To add further depth and richness to the dataset, the qualitative survey will be supplemented with ~60-minute, one-to-one semi-structured interviews with eight (n=8) participants from the intervention arm. The sample will be determined using a purposive sampling strategy, whereby the research team will identify and select information-rich cases related to the topic of interest (i.e., experiences and perspectives of MOVE SMART). Key criteria for selecting participants will include demographics, experiences shared via the qualitative survey, availability and willingness to participate, and the ability to communicate views and experiences in an articulate, expressive, and reflective manner. These interviews will allow for elaboration and clarification of the survey findings, as well as holding the potential to provide new understandings of the MOVE SMART user experience.[22] The data generated through these interviews will be analysed in accordance with the RTA approach described above, inclusive of inviting our PPI network to reflect and comment on our developing thematic interpretations.

Phase two: Will investigate the participants' experiences and acceptability of the RCT. Every individual from both the control group (n=30) and intervention group (n=30) will be invited to complete an online, qualitative survey following completion of the 24-week trial (during week-25). The survey will comprise a series of questions relating to the participants' experiences of taking part in the research, including the impact/effect of group allocation, recruitment and retention strategies, participant facing documentation, trial protocol, monitoring tools and questionnaires, and interaction with the research team. The survey distributed to the intervention arm will also incorporate a series of open-ended questions about their independent physical behaviours during weeks-13-24 of the trial to assess if, how, and why they have, or have not, continued to adopt a similar lifestyle pattern to the one prescribed in the MOVE SMART intervention. Finally, participants will be asked whether they wish to participate in a follow-on one-one semi-structured interview with a member of the research team (see below). Members of our PPI network have contributed to the development of the survey by iteratively reviewing and providing feedback on draft versions. The survey data will be analysed through RTA and in consultation with our PPI network. Following the collection and analysis of survey data, a purposeful sample of participants from the intervention group (n=8) and control group (n=8) will partake in ~60-minute, one-to-one semi-structured interviews. Key criteria for selecting interview participants will include demographics, experiences shared via the qualitative survey, availability, and willingness to discuss their experiences in detailed and expressive ways. These interviews will add richness, depth, and nuance to the dataset, providing an

opportunity for the participants to build on, explain, or challenge the thematic interpretations generated through the qualitative survey. The interview data will be analysed through the six-steps of RTA. Preliminary interpretations will be shared with, and reflective comments invited from, our PPI network.

Through conducting a rigorous qualitative exploration and analysis of the participants' experiences of MOVE SMART and the feasibility RCT, Workstream [2] will determine the acceptability of the MOVE SMART trial and identify key areas for intervention refinement.

Workstream [3]: Finalise design of the MOVE SMART RCT.
The purpose of Workstream [3] is to investigate the feasibility of progression to a definitive multi-centre RCT and to highlight any potential issues that may need to be addressed to improve delivery. Specifically, feasibility outcomes of interest include recruitment of participants, completeness of outcome measures, and adherence to the intervention MOVE SMART. These will be assessed in each arm separately, as there may be systematic differences between them; for example, those randomised to the control arm may be less likely to remain engaged than those randomised to the MOVE SMART arm.

Analysis will be by the intention-to-treat principle as far as is practically possible. All analyses will be descriptive, focussed on assessing the criteria for deciding whether to progress to a full trial. All estimates of proportions will be presented with 95% confidence intervals. Rates of recruitment and attrition will be presented for participants, along with the proportion of MOVE SMART interventions which are successfully delivered. The proportion of missing data in the proposed trial outcome measures will be assessed.

The decision to progress to full trial will be based on a traffic light system with the feasibility objectives and measures used as pre-defined stop/amend/go criteria as specified in the Table below (Table 1).

Criteria meeting all "go" (green) outcomes would lead to progression to a full RCT and no or minor revisions would be required. However, if criteria meet "amend" (amber) targets, reasons for this will be investigated with an aim to identify aspects amenable to change. One or more amber outcome would suggest that alterations or adaptations to the trial protocol, assessments, or intervention, would be required. These, in turn would be supported by the qualitative work stream and discussed with the Project Management Group (PMG). If criteria meet "stop" (red) targets, reasons will be analysed and discussion within the PMG and with independent oversight committees as major alteration would be required before conducting a full RCT. If it is determined that these rates cannot be improved, then a full trial will not be recommended.

Table 1: *MOVE SMART feasibility measures and progression criteria.*

Criterion	Critical feasibility outcome	Green	Amber	Red
(1) Recruitment rate	Feasibility of being able to recruit 60 participants within a 12-month window	≥ 75%	50-74%	< 50%
(2) Retention rate	Percentage of participants remaining in the study until completion (24-weeks total)	≥ 75%	50-74%	< 50%
(3) Clinical assessments	Percentage of participants with available data on clinical assessments	≥ 75%	50-74%	< 50%
(4) Capillary-blood self-collection	Percentage of participants returning capillary-blood home-collection kits	≥ 75%	50-74%	< 50%
(5) Adherence to intervention	Percentage of participants completing the MOVE SMART intervention	≥ 75%	50-74%	< 50%
(6) Intervention and trial protocol	Percentage of participants report positive experiences (acceptability) and adherence to intervention components (feasibility)	≥ 75%	50-74%	< 50%
(7) Safety of intervention	Monitoring and review of intervention related serious adverse events (SAEs). The TSC will oversee SAEs across the intervention and control arms. GO: When the rates of intervention-related SAEs are low and the TSC is happy for continuation to a definitive trial. STOP: When the rates of intervention-related SAEs are considered unacceptably high, or when any single SAE indicates an unacceptable risk to participants.			

We will finalise the intervention specification costs, and research procedures based on our feasibility and acceptability testing.

5.3 End of study
The end of study will be on completion of: follow-up monitoring for study participants, data collection, data analysis, the completion of sample processing/analysis and when the design of the subsequent RCT has been finalised.

All participants (control or intervention) will have access to the intervention once the research has finished.

6) STUDY PARTICIPANTS

6.1 Inclusion Criteria:

-Patients with Type 1 chronic plaque psoriasis (disease onset before age 40 years) with/without stable psoriatic arthritis
-Aged 18-60 years (due to the prevalence of CVD disease in the >60s in the UK, which means that we are likely to detect greater differences in CVD risk in those <60)
-Stable disease but measurable residual psoriasis/psoriatic arthritis
-No treatment/dose changes for 2-months
-High sedentary time (irrespective of level of physical activity): defined using the Short Form International Physical Activity Questionnaire (IPAQ) as >8 hours per day.

6.2 Exclusion Criteria:

-Unable to rise from a sitting to a standing position, walk around a room (independently or aided by a walking stick, frame or trolly) whilst maintaining a steady pace (~30 steps/minute) for 2-minutes without stopping.
-Worsening inflammatory arthritis
-Presence of other significant comorbid conditions that would interfere with the ability to participate in physical activity or the study protocol, such as severe cardiovascular disease, severe chronic obstructive pulmonary disease (COPD).
-Regular engagement in HIGH levels of physical activity (as defined by IPAQ) whilst spending MINIMAL time in sedentary activities during day-waking hours (as calculated by IPAQ, <6 hours per day).

6.3 Recruitment:

We will conduct a decentralised, randomised controlled feasibility trial of 60 participants recruited from across the UK and supported by the national reach of our partner organisations - the UK Psoriasis Association and the UK Dermatology Clinical Trials Network (UK DCTN). Our decentralised study will utilise a digitally enabled remote trial delivery model. If feasible, we plan to utilise this approach in our definitive trial.

Recruitment (UK-wide): The UK Psoriasis Association will advertise the study through their online, mailing and journal/newsletter platforms.
We will also advertise the study through the UK Dermatology Clinical Trial Network (DCTN) through their online, mailing and journal/newsletter platforms.
There will also be advertising within the Psoriasis Clinic at Salford Royal Hospital (led by the Lead Applicant) which caters for psoriasis referrals from primary and secondary care across North-West England. No access to medical records is required.

Recruitment to the study will therefore be through self-referral of interested individuals directly to the study team.

Following an expression of interest in learning more about the study, potential volunteers will receive a written information sheet. They will have the opportunity to ask questions about the study will be able to discuss the study with the research team. Volunteers will be given as much time as they need to consider whether they wish to participate in the research study. The research team will screen potential participants (virtually) to determine their suitability as per the inclusion / exclusion criteria. A screening

proforma is included with this application bundle. Informed consent will then be taken by a member of the research team (usually the PDRA who will be employed to work on this study) via the online Qualtrics platform (note: the PDRA will be with the participant virtually during the consent process).

Non-clinical members of the research team will hold research passports

No recruitment will be made from vulnerable groups.

6.4 Randomisation:

We will use a stratified randomisation procedure to ensure that participants are well-balanced between the intervention (sedentary behaviour fragmentation) and control groups and are matched based on age and sex (all participants will have psoriasis).

Randomisation Procedure:

1. **Stratification by Key Variables:**
   Age Groups: Defined as follows (18-30, 31-45, 46-60).
   Sex: Male/female participants.

2. **Create Stratified Blocks:**
   For each combination of age group and sex, we will create the following separate blocks:
   Males aged 18-30
   Females aged 18-30
   Males aged 31-45
   Females aged 31-45
   Males aged 46-60
   Females aged 46-60

3. **Block Randomise within Each Stratum:**
   We will randomly allocate participants within each stratum to either the **treatment group (MOVE SMART intervention)** or the **control group**. This will ensure balance at all points during recruitment. Block sizes will be fixed (at 4 or 6) to ensure a balanced number of participants in each group across strata.

The study will effectively be open label.

6.5 Participants who withdraw consent [or lose capacity to consent]:

Participants can withdraw consent at any time without giving any reason, as participation in the research is voluntary, without their care or legal rights being affected.

Data and biological samples will be pseudonymised. It will not be possible to remove samples / data from the project once the pseudonymised data key has been destroyed, which will anonymise these data. These data will then form part of the data set.

Participants in this study are unlikely to lose capacity during course of the study. Therefore, the research team does not propose to monitor capacity nor will the clinical team be doing this for us. Therefore, continued capacity will be assumed.

7) OUTCOME MEASURES

It is envisaged that this study will describe the following outcomes:
-determine feasibility of the research processes (recruitment rate, willingness of eligible participants to be randomised, retention rate, reasons for loss to follow-up) and delivery of the intervention (fidelity of adherence with MOVE SMART, identify potential adverse effects and test collection of clinical and health-related outcome measures)

-determine acceptability of the intervention using a qualitative, mixed-methods approach

-finalise the design of the future multi-centre RCT

-inform decisions about primary and secondary outcomes for the RCT and associated sample size calculations.

8) DATA COLLECTION, SOURCE DATA AND CONFIDENTIALITY

Personal data

Identifiers including name and address will be collected. These are required in order to contact participants and arrange delivery of study materials / equipment during the study. No individuals outside the research team will have access to personal data.

With consent from participants contact details will be retained to permit contact about other research projects in the future and to inform them of the research outcomes. We will therefore store the contact details of participants securely in digital format on University of Manchester Research Data Storage Service.

Contact with the participant's GP will only be with participant consent.

Data storage

Data will be stored by using the University of Manchester Research Data Storage Service. Data access will be controlled to ensure data security. This will be managed by using the RDM service and include password protection.

Identifiable personal data stored on manual files will be securely located in locked University of Manchester offices on the Salford Royal Hospital site.

Research data

The data will be analysed at The University of Manchester. Only members of the research team will generate, handle and analyse the data.

Collaborators from other institutions will be registered with The University of Manchester IT Services as visitors, after which they too will be granted access to the RDM service via the CI (Dr Helen Young).

Only pseudonymised data will be used for research data analysis. The pseudonymised data key will be stored separately from the dataset. Only members of the research team will generate, handle and analyse the data. Once all of the data has been analysed, we will destroy the key, anonymising these data.

Study data and material may be looked at by individuals from The University of Manchester, from regulatory authorities or from the NHS Trust, for monitoring and auditing purposes, and this may well include access to personal information.

With participant consent fully anonymised research data will be shared in order to support additional research in accordance with The University of Manchester's Research Privacy Notice.

Collection of data:

Self-completion questionnaires and medical history forms will be completed online using the Qualtrics platform which is approved by The University of Manchester. Participants will be supplied with their study ID, by the research team, prior to completion of the Qualtrics forms. Participants will be reminded to not supply / include any personal data which will identify them in any free text questions.

Virtual contact with research participants:

All research activities will be conducted virtually; on an approved University of Manchester platform. However, with the exception of the one-one semi-structured interviews (below), these interactions will not be recorded or transcribed.

We request permission for participants to meet the study team virtually to complete their functional capacity assessments. These are validated measures of functional capacity and can be easily demonstrated and conducted remotely. These data may be recorded (on approved University of Manchester devices).

Virtual platforms will also be used to conduct the one-one semi-structured interviews with research participants in Workstream [2]. For these recording will include both voice (audio) and face (video). However, participants can, if they prefer, disable their camera so that only their voice is recorded. The recording of the conversation will be used to make a transcript and once correct, the recording will be deleted. Any information from the transcript that could identify a participant will be removed. The transcript will be made by a member of the University of Manchester study team. Importantly, the transcription will happen on University of Manchester premises and the member of staff will have signed a confidentiality agreement. **Publication of direct quotations from respondents will not include information which could allow identification of individuals.**

Using virtual platform software may mean that personal data is transferred to a country outside of the European Economic Area, some of which have not yet been determined by the United Kingdom to have an adequate level of data protection. Appropriate legal mechanisms to ensure these transfers are compliant with the Data Protection Act 2018 and the UK General Data Protection Regulation are in place. The recordings will be removed from the above third-party platform and stored on The University of Manchester managed file storage as soon as possible following the completion of data collection.

Capillary blood samples:
Finger-prick capillary blood will be collected by volunteers at home and returned (via mail) directly to the research team at The University of Manchester.

Initially, samples will be temporarily stored in the Dermatology laboratory, at Salford Royal Hospital, which is a University of Manchester facility. Access at this location is restricted (via key fob) and a keypad code is required to access the laboratory area.

Samples will then be transferred for storage in the Dermatology laboratories at The University of Manchester, on main campus (Stopford Building). This will be done under an OID agreement. Access to the Stopford Building itself is restricted and a swipe card (issued by the laboratory manager) is required for entry to the Dermatology laboratory. Only the research team will have access to the samples.

Analysis of the samples will take place in the Dermatology Laboratories.

The samples are likely to be fully utilised during laboratory analysis, however, should there be residual material this will be disposed of as per the Human Tissue Act Code of Practice on the Removal, Storage and Disposal of Human Organs and Tissue at the end of the study.

End of the study
The University of Manchester guidance is that personal data (including consent forms) should be retained for 7 years following the end of the study and that research data based on clinical samples or relating to public health should be retained for 15 to 20 years. Residual human tissue samples will be destroyed at the end of the study.

Archiving will be in line with The University of Manchester records retention schedule. Paper documents will be stored via Iron Mountain off-site storage. Digital records will be archived via the research data archive service offered, centrally-hosted and administered, by The University of Manchester. This archive service is recommended by University of Manchester Research Data Management (RDM) Service as it satisfies the Research Council UK RDM guidelines.

Paper records which contain personal and research data will be shredded once they are no longer required. This will be performed by the University of Manchester's confidential paper shredding and disposal service. Electronic records will be erased with support from The University of Manchester IT Support Services who will arrange for it to be destroyed.

At the end of the project, we will deposit a fully anonymised dataset (including de-identified interview transcripts) in an open data repository where it will be permanently stored. We will use an approved University of Manchester online repository such as: Figshare at the University of Manchester Library. Researchers at other institutions and others will have access to the anonymised data directly from the repository and use it for further research or to check our analysis and results.

Data custodian
Dr. Helen Young, the CI for this study, will be responsible for the data.

9) STATISTICAL CONSIDERATIONS

9.1 Statistical Analysis

To interrogate our quantitative data: if the data is normally distributed the paired t-test will be used to compare the data. The paired samples Wilcoxon test (also known as Wilcoxon signed-rank test) will be used as a non-parametric alternative to paired t-test if the data is not normally distributed.

For our qualitative outcomes: data will be analysed using Reflexive Thematic Analysis (RTA), which involves six iterative phases: familiarising yourself with the dataset; coding; generating initial themes; developing and reviewing themes; refining, defining, and naming themes; and writing up. RTA is widely used in sport, health, and exercise research, providing a robust method for developing deep and compelling understanding of patterned meaning across a qualitative dataset. As part of our RTA approach, findings from the survey will be shared with our PPI networks and comments invited regarding our developing thematic interpretations.

9.2 Sample Size:

Our sample size (n=60, equally divided between 2 groups) is calculated based on our quantitative traffic light progression criteria. A sample size of 30 per group will give at leave 80% power to reject a true proportion being in the red zone (< 50%) if we observe a proportion in the green zone (⩾ 75%; ***Table 1 / Protocol section 5.2***).

10) MONITORING AND QUALITY ASSURANCE

The responsibility for monitoring the study is delegated to the CI – Dr Helen Young and is subject to the audit and monitoring regimen of The University of Manchester (sponsor).

In addition, the study will be run on a day-to-day basis by the Project Management Group (PMG), comprising the Lead and Co-Investigators for the study. The PMG will report to a Trial Steering Committee (TSC), who will review the progress of the trial and report on this to the funder (NIHR). The TSC will also fulfil the role of the Data Monitoring and Ethics Committee (DMEC). The TSC will review the progress of the trial and perform an interim analysis at least annually and will also provide advice on the conduct and safety of the trial. Observers from the NIHR will be invited to attend all TSC meetings.

11) SAFETY CONSIDERATIONS AND ADVERSE EVENTS

The CI is responsible for:

- identifying, recording and reporting adverse events (AEs)
- assessing adverse events and adverse reactions as per the protocol
- reporting SAEs and urgent safety measures to the sponsor and REC as per the requirements of the Research Ethics Service

- maintaining a log of adverse events occurring during the study and making this available to the Sponsor upon request.

Participation in this study involves minimal/minor risks, which are well-managed through the provision of appropriate training, support, and adherence to data privacy regulations. There are no potential risks for the researchers themselves. However, the study involves a novel intervention together with study procedures for which there is no established risk profile. On this basis all AEs will be recorded and reported to the CI – Dr Helen Young during the study (from recruitment to completion of all study procedures at week-25).

AEs will be identified by specifically asking all participants about AEs at each point of contact with the study team. A dated case report form (CRF) detailing a description of the AE will be used and reported immediately to the CI – Dr Helen Young.

AEs will be assessed for seriousness, relatedness, expectedness and severity by the CI – Dr Helen Young.

It is noted that from a regulatory perspective, a serious adverse event (SAE) or serious adverse reaction (SAR) is one that:

- results in death;
- is life-threatening;
- requires hospitalisation or prolongation of existing hospitalisation;
- results in persistent or significant disability or incapacity;
- consists of a congenital abnormality or birth defect;
- is otherwise considered medically significant by the investigator.

Note: Non-serious AEs/ARs for this study do not need to be reported to the sponsor.

All SAEs for the study will be reported to the sponsor, by the CI, via FBMHethics@manchester.ac.uk within one working day of being notified of the SAE.

Any SAEs that are considered related to study intervention/procedures and unexpected (ie. an unexpected SAR) will be reported to the REC within 15 days of the CI first becoming aware of the event.

Where urgent safety measures are required (ie. steps taken by the CI and/or research team in the event that there is an immediate risk to a participant or participants, without the prior approval of the NHS REC/HRA), the CI will notify the REC immediately by telephone and then follow-up with a substantial amendment within 3 days outlining the measures that have been taken and why. A copy of the amendment will be submitted to FBMHethics@manchester.ac.uk before being submitted to the REC, making it clear that it is being submitted to the REC as part of implementing an urgent safety measure.

In addition, oversight of safety monitoring will be performed by the Trial Steering Committee (TSC) who will fulfil the role of the Data Monitoring and Ethics Committee (DMEC). The trial will STOP: when the rates of intervention-related SAEs are considered unacceptably high, or when any single SAE indicates an unacceptable risk to participants.

12) PEER REVIEW

This proposal was extensively reviewed during the NIHR grant application process (2 x stages) to the NIHR Research for Patient Benefit programme by at least two independent external reviewers.

13) ETHICAL and REGULATORY CONSIDERATIONS

13.1 Approvals

NHS Research Ethics Committee and Health Research Authority (HRA) approvals will be obtained before commencing research.

The study will be conducted in full conformance with all relevant legal requirements and the principles of the Declaration of Helsinki, Good Clinical Practice (GCP) and the UK Policy Framework for Health and Social Care Research 2017.

Clinical Trial Authorisation from the MHRA is not required as the study is designated as clinical research not involving investigational medicinal products or medical devices.

The study will be registered with the International Standard Randomised Controlled Trial Number (ISRCTN) registry.

13.2 Risks

Risks to participants:
Participation in this study involves minimal/minor risks, which are well-managed through the provision of appropriate training, support, and adherence to data privacy regulations. Potential risks and mitigation strategies include:

- Physical Activity-Related Risks: MOVE SMART involves light-intensity physical activity. There is a small risk of minor discomfort or injury, especially for participants with pre-existing conditions. To mitigate this, participants can choose activities that suit their comfort level, and comprehensive guidance will be provided via an illustrated booklet to ensure safe execution of movements.
- Capillary Blood Collection: Participants will self-collect capillary blood at home using a finger-prick method. This may cause minor discomfort or bruising. To minimize risks, participants will receive training and supplies for safe collection. Support will be available for any concerns during the process.
- Use of Technology: Participants will use a mobile app to receive prompts and log activities. Digital literacy challenges may arise, particularly for those less familiar with smartphones. Training and technical support will be provided, and devices will be supplied to those without access, ensuring that all participants can engage comfortably with the study.

Risks to researchers:
There are no potential risks for the researchers themselves.

14) STATEMENT OF INDEMNITY

The University has insurance available in respect of research involving human subjects that provides cover for legal liabilities arising from its actions or those of its staff or supervised students. The University also has insurance available that provides compensation for non-negligent harm to research subjects occasioned in circumstances that are under the control of the University.

15) FUNDING and RESOURCES

This study is funded by a grant from the NIHR Research for Patient Benefit (RfPB) Programme: NIHR207157:

All equipment / devices used in this study will be purchased by the study team (for use in this study) or are already owned by the study team.

At the end of the study the following equipment provided for participant use: resistance bands, body-weight scales and blood pressure monitors will be retained by the participant. GeneActive accelerometers and any smartphones / tablets provided by the research team for participant use will remain the property of the research team and will need to be returned (by participants) to the research team.

16) PUBLICATION POLICY

It is our usual practice to send (anonymised group) results to all study participants, unless they have opted out. We will, in addition, produce a patient-centred summary of progress at the mid-point of the study

to keep participants informed. We will hold an information dissemination event at the end of the study for individuals with lived-experience, third sector organisations and health professionals. With our PPI advisors we will co-design an infographic summary, to be distributed via email, social media and on partner third sector and professional organisation websites.

We will publish the results in the highest impact peer-reviewed “open access” journals achievable such as general medical (Lancet/NEJM), dermatology (BJD/JID) and Sports and Exercise Science journals.
We believe our work will generate widespread interest, well beyond the immediate fields of dermatology, inflammation, and Sport and Exercise Science/Medicine and will present the study results at national and international dermatology Sport and Exercise Science conferences.

The study will also report via the International Standard Randomised Controlled Trial Number (ISRCTN) registry.

17) REFERENCES

1. **Ebrahim S, et al.** Carotid plaque, intima media thickness, cardiovascular risk factors, and prevalent cardiovascular disease in men and women: the British Regional Heart Study. Stroke. 1999;30:841-50.
2. **UKDCTN commissioned national survey of Standard Care for patients with psoriasis.** 2023.
3. **Sheppard R, et al.** Can an aerobic exercise intervention for patients with psoriasis improve health outcomes and support lifestyle behaviour change? Br J Dermatol. 2023;188:iv168.
4. **World Health Organisation.** Global recommendations on physical activity for health. 2014
5. **Grant D, et al.** Minimizing sedentary behavior (without increasing medium-to-vigorous exercise) associated functional improvement in older women is somewhat dependent on a measurable increase in muscle size. Aging (Albany NY). 2020;12:24081-100.
6. **Grant D, et al.** The effects of displacing sedentary behavior with two distinct patterns of light activity on health outcomes in older adults (implications for covid-19 quarantine). Front Physiol. 2020;11:574595.
7. **Onambele-Pearson G, et al.** Influence of habitual physical behavior - sleeping, sedentarism, physical activity - on bone health in community-dwelling older people. Front Physiol. 2019;10:408.
8. **Diaz KM, et al.** Patterns of sedentary behavior and mortality in U.S. middle-aged and older adults: A national cohort study. Ann Intern Med. 2017;167:465-75.
9. **Grant D, et al.** Displacing sedentary behaviour with light intensity physical activity spontaneously alters habitual macronutrient intake and enhances dietary quality in older females. Nutrients. 2020;12:2431.
10. **Takemoto M, et al.** Participants' Perceptions on the Use of Wearable Devices to Reduce Sitting Time: Qualitative Analysis. JMIR Mhealth Uhealth. 2018;6:e73.
11. **Cordingley L, et al.** Identifying and managing psoriasis-associated comorbidities: the IMPACT research programme. Southampton (UK): NIHR Journals Library; 2022.
12. **Henry AL, et al.** The relationship between sleep disturbance, symptoms and daytime functioning in psoriasis: A prospective study integrating actigraphy and experience sampling methodology. Sleep Med. 2020;72:144-9
13. **Sheppard R, et al.** Developing an aerobic exercise intervention for patients with psoriasis to support lifestyle behaviour change and improve health outcomes. Clin Exp Dermatol. 2023;48:5-11.
14. **Ansari S, et al.** The use of whole blood capillary samples to measure 15 analytes for a home-collect biochemistry service during the SARS-CoV-2 pandemic. Ann Clin Biochem. 2021;58:411-21.
15. **Auker L, et al.** What are the barriers to physical activity in patients with chronic plaque psoriasis? Br J Dermatol. 2020;183:1094-1102.
16. **Auker L, et al.** Physical activity is important for cardiovascular health and cardiorespiratory fitness in patients with psoriasis. Clin Exp Dermatol. 2022;47:289-96.
17. **Ryan DJ, et al.** The difference in sleep, sedentary behaviour, and physical activity between older adults with 'healthy' and 'unhealthy' cardiometabolic profiles: A cross-sectional compositional data analysis approach. Eur Rev Aging Phys Act. 2019;16:25.
18. **Braun V, et al.** The online survey as a qualitative research tool. Int J Soc Res Methodol. 2021;24:641-54.
19. **World Health Organisation.** *Classification of digital health interventions v1.0: a shared language to describe the uses of digital technology for health*. https://apps.who.int/iris/handle/10665/260480).
20. **Braun V, et al.** Thematic analysis: A practical guide. Thousand Oaks: Sage Publications Ltd. 2022.
21. **Trainor LR, et al.** Developing the craft: Reflexive accounts of doing reflexive thematic analysis. Qual Res Sport Exerc Health. 2021;13:705-26.
22. **McGannon KR, et al.** Qualitative research in six sport and exercise psychology journals between 2010 and 2017: An updated and expanded review of trends and interpretations. Int J Sport Exerc Psychol. 2021;19:359-79.
