# Supplementary figures and images for: Study protocol for a feasibility randomised controlled trial of MOVE SMART—An intervention to increase physical activity, reduce sedentary behaviour and improve health outcomes in patients with psoriasis
Source: PLoS One. 2026 Mar 16;21(3):e0343922. doi: 10.1371/journal.pone.0343922 (PMC12991228; doi:10.1371/journal.pone.0343922)

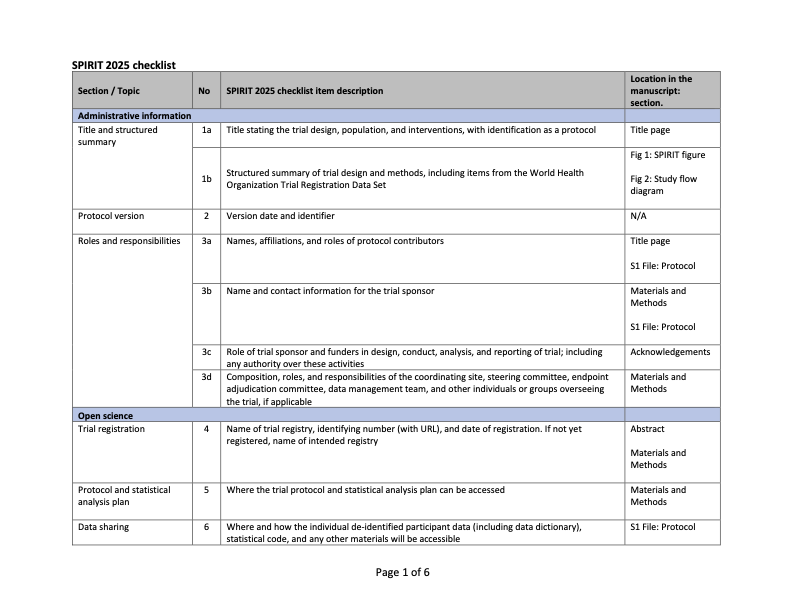

Supplement: S1 Fig — (TIFF) [file pone.0343922.s002.tiff]

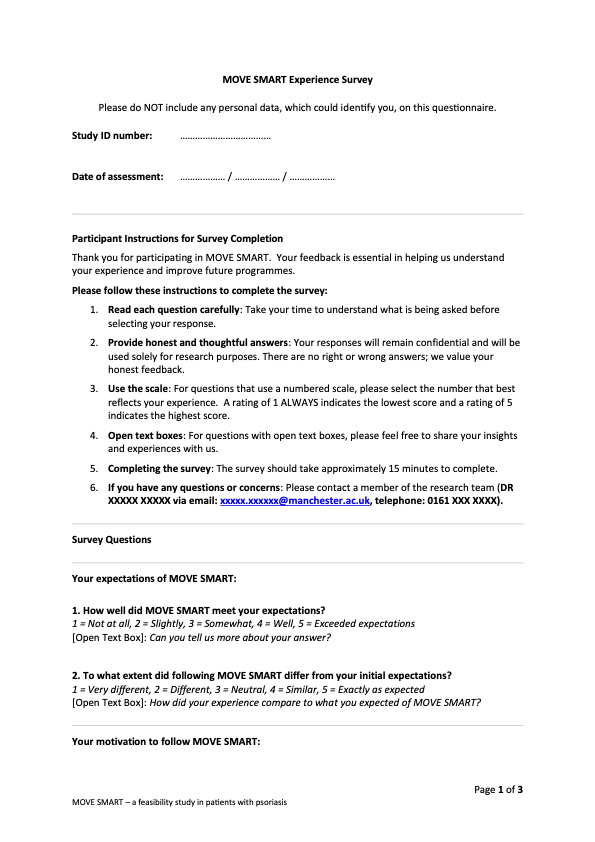

Supplement: S2 Fig — (TIFF) [file pone.0343922.s003.tiff]

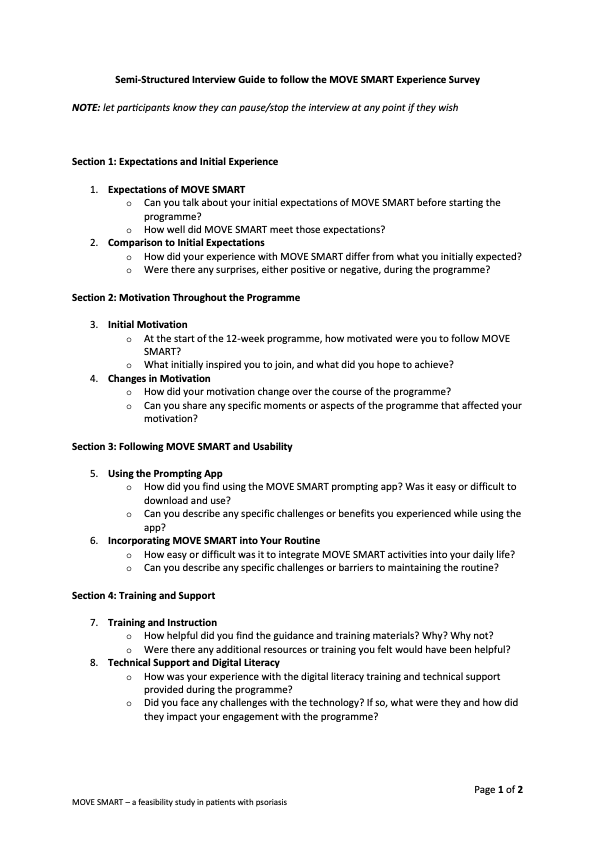

Supplement: S3 Fig — (TIFF) [file pone.0343922.s004.tiff]

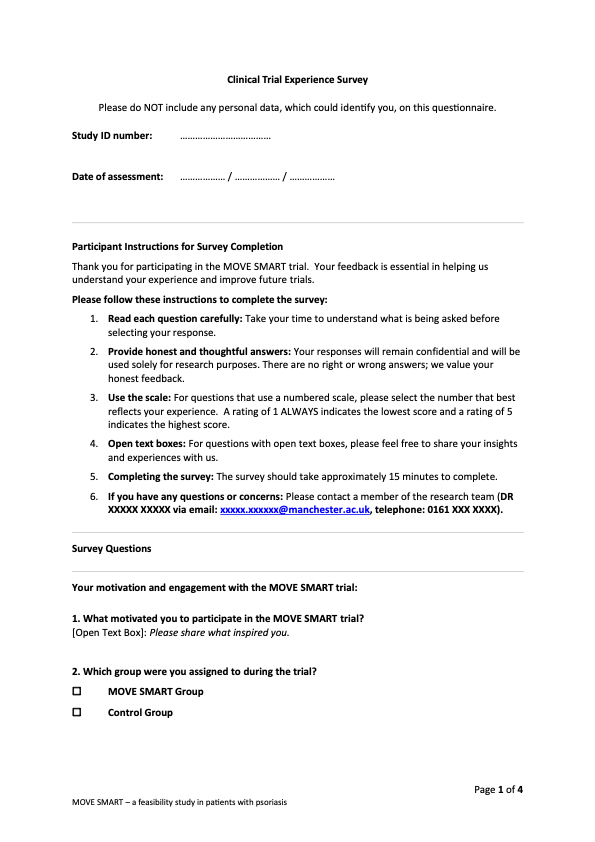

Supplement: S4 Fig — (TIFF) [file pone.0343922.s005.tiff]

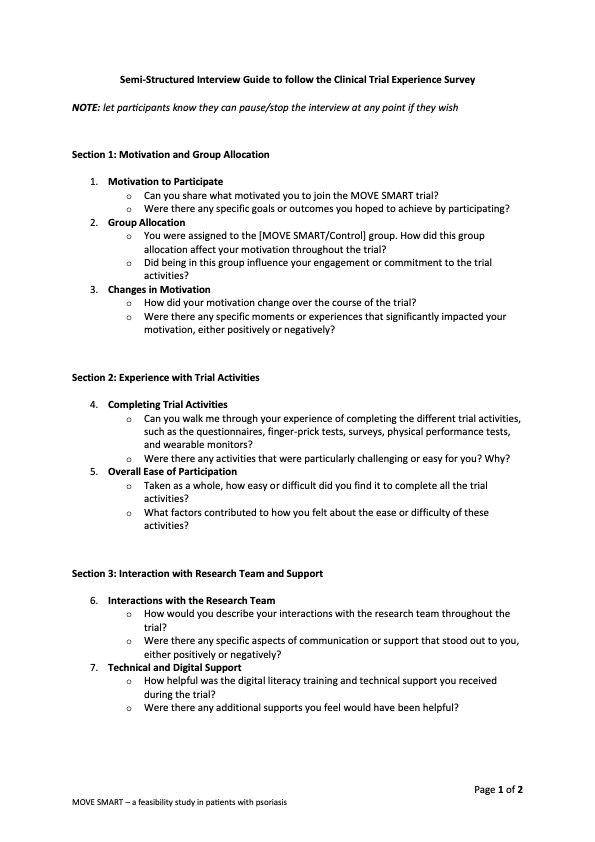

Supplement: S5 Fig — (TIFF) [file pone.0343922.s006.tiff]
